# Impacts of iodine factory effluents on the water quality and ecosystem health of Incheh Lagoon in Iran

**DOI:** 10.1038/s41598-026-52359-0

**Published:** 2026-05-16

**Authors:** Rasoul Ghorbani, Rahman Patimar, Seyed Abbas Hosseini, Abdol Azim Fazel, Fatemeh Abbasi, Aliakbar Hedayati, Tahereh Bagheri, Parisa Maleki, Meysam Salarjazi, Arsalan Bahalkeh, Ali-Naghi Maghsoudlou

**Affiliations:** 1 Gorgan University of Agricultural Sciences and Natural Resources, Gorgan, Iran; 2 Gonbad Kavous University, Gonbad Kavous, Iran; 3 Iranian Fisheries Science Research Institute, Tehran, Iran

**Keywords:** Heavy metals, Industrial effluents, Water pollution, Wetland ecosystems, Incheh Lagoon, Ecology, Ecology, Environmental sciences

## Abstract

Industrial effluents containing heavy metals pose a growing threat to aquatic ecosystems, especially in ecologically sensitive wetlands such as the Incheh lagoon in north- east Iran. In this study, physico- chemical parameters, heavy metal concentrations, and water quality of wastewater from a local iodine production plant discharged into the lagoon are comprehensively analysed. The concentration of 30 heavy metals were measured using flame atomic absorption spectrophotometry (FAAS) and standardised analysis protocols. In particular fluoride (1.36–4.6 mg/L) and manganese (2.25–3.89 mg/L) exceeded the limits set by the US Environmental Protection Agency (USEPA, 2017) and the Water Resources Commission (WRC, 2003). Although Artemia species were present in the lagoon, their short residence time and relatively low metal accumulation compared to FAO/WHO guidelines indicated minimal risk to migratory bird populations. Uncontrolled drainage channels have been identified as the main source of contamination. To tackle this problem, the study recommends joint actions involving forest authorities, municipal bodies and local stakeholders to introduce sustainable watershed management practices.

**Supplementary Information:**

The online version contains supplementary material available at 10.1038/s41598-026-52359-0.

## Introduction

Iodine (I, atomic number 53) is a vital micronutrient required for the regulation of metabolic processes in both humans and animals^1^. Due to its physiological importance, iodine is listed among the World Health Organization’s essential medicines^2^. While iodine deficiency continues to pose a global health challenge, excessive environmental exposure particularly through contaminated water sources has emerged as a growing concern in certain regions^3^. Chile and Japan currently dominate global iodine production, collectively generating approximately 30,000 tons annually (SQM Iodine^4^. However, the extraction and processing of iodine may contribute to localized environmental impacts, especially in sensitive aquatic ecosystem. Despite the global importance of iodine production, the environmental consequences of iodine-related effluents, particularly in wetland ecosystems, remain insufficiently documented, creating a clear knowledge gap.

Wetlands, defined as transitional ecosystems that are permanently or seasonally inundated, play a critical role in maintaining ecological balance. They regulate hydrological cycles, purify water, and provide habitat for diverse flora and fauna^5,6^. NOAA (2018) emphasizes the essential function of wetlands in maintaining ecosystem balance, such as regulating flooding, purifying water, and supporting wildlife. However, their capacity to absorb excess nutrients, sediments, and pollutants also makes them vulnerable to contamination. Recent studies have demonstrated that wetlands increasingly act as sinks for heavy metals, accumulating toxic substances from industrial runoff and atmospheric deposition^7–9^. This dual role both as ecological protectors and pollution reservoirs underscores the need for comprehensive environmental assessments in wetland systems. Nevertheless, the specific impacts of iodine extraction wastewater on wetland biogeochemistry have not been systematically evaluated.

The global increase in heavy metal contamination represents a critical threat to both environmental integrity and human health. Individuals are exposed to these persistent toxicants through various pathways, including ingestion of contaminated water and food, inhalation of airborne particles, and dermal contact. These exposoures are primarily driven by industrialization, urbanization, and agricultural runoff^10,11^. Among the most hazardous heavy metals, lead (Pb), cadmium (Cd), and manganese (Mn) are consistently ranked as high-priority pollutants due to their widespread occurrence and potent toxicity^12,13^. Even at low concentrations, these metals can disrupt biological systems and accumulate in vital organs. Lead exposure has been linked to neurological, renal, cardiovascular, and reproductive dysfunctions, while cadmium is associated with kidney damage, hypertension, and carcinogenesis. Manganese, although essential in trace amounts, can cause neurotoxicity and oxidative stress when present in excess^9,14,15^. Recent epidemiological studies have demonstrated synergistic toxic effects when individuals are exposed to mixtures of these metals, exacerbating risks of hypertension, stroke, and cognitive decline^16,17^. Wetlands, due to their sediment-trapping capacity and biological productivity, often serve as sinks for heavy metals. These contaminants can bioaccumulate in aquatic organisms and enter the human food chain, posing long-term health risks. The ingestion of contaminated fish, crustaceans, or algae facilitates the transfer of metals to humans, where they may impair the central nervous system, lungs, kidneys, liver, endocrine glands, and skeletal system^11,18^. Understanding these exposure pathways is essential for developing effective environmental monitoring and public health interventions. Given these risks, evaluating heavy metal dynamics in wetlands affected by industrial effluents is crucial for both ecological and human health protection.

Wetlands are dynamic aquatic systems influenced by a combination of natural and anthropogenic factors. Natural drivers such as wind patterns, seasonal runoff, and geomorphological variations interact with human-induced pressures including land-use changes, industrial discharge, and pollution to shape wetland ecosystems^19^. The extent of these impacts is modulated by physical attributes such as basin geometry, number of inlets, and hydrodynamic parameters like depth and width^20^.

In aquatic environments, trace metals typically exhibit low solubility and tend to accumulate in sediments, where they pose long-term ecological risks^21,22^. Sediment-bound metals can be remobilized under changing environmental conditions, leading to increased bioavailability and toxicity in benthic and pelagic organisms. Geochemical tools including pollution indices, isotopic tracing, and speciation analysis are essential for identifying contamination sources and assessing ecological risks^23,24^. These methods enable the creation of spatial and temporal databases that support long-term monitoring and remediation planning. While some heavy metals originate from natural processes such as rock weathering and volcanic activity, anthropogenic sources particularly mining, agriculture, and industrial operations are the dominant contributors to surface contamination^22,25^. Understanding the interplay between natural and human drivers is critical for managing wetland ecosystems and mitigating the adverse effects of metal pollution. However, no prior study has investigated how iodine extraction wastewater influences these geochemical processes in lagoon environments.

The presence and proliferation of heavy metals in wetland environments can significantly disrupt ecological functions and threaten aquatic biota, with cascading effects on human health^26^. Essential trace elements such as copper (Cu), chromium (Cr), zinc (Zn), iron (Fe), and manganese (Mn) play critical roles in metabolic processes, yet elevated concentrations can induce oxidative stress and cellular toxicity in aquatic organisms^27^^[,8^. Moreover, toxic metals including arsenic (As), cadmium (Cd), lead (Pb), mercury (Hg), and chromium (Cr) are classified as priority pollutants due to their persistence, bioaccumulative nature, and adverse health impacts^8,26,28,29^. Given the ecological sensitivity of lagoon systems, assessing the extent of heavy metal contamination in regions receiving industrial effluents is essential.

It is therefore imperative to conduct a comprehensive assessment of heavy metal concentrations in lagoon ecosystems. These contaminants pose acute and chronic risks to exposed organisms, particularly those at higher trophic levels. Humans and apex predators are especially vulnerable to bioaccumulation effects, which can lead to neurological, reproductive, and immunological disorders^8,30,31^. Studies on trophic transfer and biomagnification are essential, as they reveal how contaminated prey can transmit toxic metals to predators, ultimately disturbing ecological equilibrium and endangering public health^26,32^. Despite these concerns, the ecological consequences of long-term iodine-related effluent discharge into the Incheh Lagoon have not been previously quantified.

The Incheh Lagoon supports a unique ecological community, including cyanobacteria and *Artemia parthenogenetica*, which serve as critical winter food sources for migratory flamingos. The lagoon’s hydrological dynamics have fluctuated significantly due to variable precipitation and regulated discharges from the Voshmgir Dam. Since 2008, following the establishment of iodine extraction facilities (Khazar Mine and Shorabeh Iodine Companies of Golestan), authorized effluent releases have entered the lagoon. However, no integrated geochemical assessment has been conducted to evaluate the long-term effects of these discharges on the lagoon’s water column. This study investigates the geochemical properties of both surface and deep-water columns in the lagoon, fifteen years after the onset of iodine-related industrial discharge. During 2023–2024, heavy metal concentrations were measured to assess contamination levels. To address the identified research gap, this study aims to: (1) quantify heavy metal concentrations in lagoon water influenced by iodine extraction effluents; (2) compare measured values with national and international standards; and (3) provide a baseline for future ecological risk assessments. Chemical speciation refers to identifying the chemical forms of metals (such as free ions or complexed species), which determine their bioavailability and potential toxicity to aquatic organisms. This clarification is essential for understanding how metals discharged from iodine extraction effluents may affect algae and *Artemia parthenogenetica* in the lagoon ecosystem.The lagoon’s distinct features such as fluctuating water levels and elevated salinity were also considered, as these factors influence metal solubility and uptake. Seasonal shifts in water chemistry can alter metal behavior, affecting absorption rates in biota. The dominance of *Artemia parthenogenetica*, known for its resilience to extreme conditions, adds complexity to the ecological assessment. Despite their tolerance, these organisms can accumulate metals in their tissues. They may potentially transfer them to higher trophic levels, including migratory birds. By integrating water and biological sampling, this research provides a comprehensive baseline of heavy metal contamination, essential for future remediation efforts and long-term ecosystem monitoring.

## Materials and methods

### Study area

Incheh Lagoon is located in northeastern Iran within the Caspian Sea basin (54°29’ to 54°42’ E and 37°9’ to 37°17’ N). The lagoon exhibits pronounced seasonal variability in morphology, hydrodynamics, and water volume, primarily governed by surface runoff originating from the Voshmgir Dam. The hydrological regime is characterized by a wet season extending from October to May and a dry season from June to September^33^. Annual precipitation averages 362 mm, while evaporation rates reach approximately 1203 mm^34^. Hydrological assessments indicate that subsurface and floodwater exchanges with the lagoon are negligible. From a geological perspective, the lagoon’s catchment area is situated a top extensive halite deposits, which substantially contribute to its elevated salinity levels. The lagoon’s water showed an average electrical conductivity of 7490 µS/cm, with a pH range between 7.5 and 8.6. The mean concentration of dissolved oxygen is 7.49 mg/L. Seasonal fluctuations influence the water depth, which varies from 0.4 to 1.2 m^34^. *Artemia parthenogenetica* is the dominant zooplankton species within the lagoon’s ecosystem. Geographically, the lagoon borders the internationally recognised freshwater bodies Almagol, Adjigol, and Alagol wetlands, which serve as important resting place for Siberian migratory birds. Prior to 2007, when effluent from a nearby iodine production facility began discharging into the lagoon, its surface area ranged between 150 and 450 hectares. By 2024, the lagoon had expanded to approximately 970 hectares, extending longitudinally from west to east. The current average depth is approximately 3.5 m, with maximum depths reaching up to 6.5 m. At present, the lagoon receives untreated industrial wastewater containing heavy metals at an estimated inflow rate of 200 L per second, posing significant ecological and environmental risks.

### Sampling collection

Water samples were collected from the lagoon during two distinct seasons: autumn 2023 and winter 2024. Sampling was conducted at three geographically distributed sites: western, central, and the newly identified eastern location, as part of a scientific research project. The lagoon is a public-access water body, and routine water sampling does not require any formal governmental permit; therefore, no special authorization was needed for sample collection. The selection of autumn and winter aimed to capture the major seasonal variability of the lagoon. Autumn represents the post‑dry period with higher evaporation and potentially elevated pollutant concentrations, whereas winter corresponds to the period of freshwater inflow and ecological recovery. These two seasons therefore provide representative hydrological and ecological conditions for assessing water quality and metal dynamics in the lagoon. At each site, water samples were collected from the surface layer (0–30 cm depth) and the near-bottom layer (20–30 cm above the sediment–water interface) to capture vertical variability in water quality parameters. Additionally, a control sample was collected from a neighboring lagoon located approximately 500 m away to facilitate comparative analysis.

**Fig. 1:**
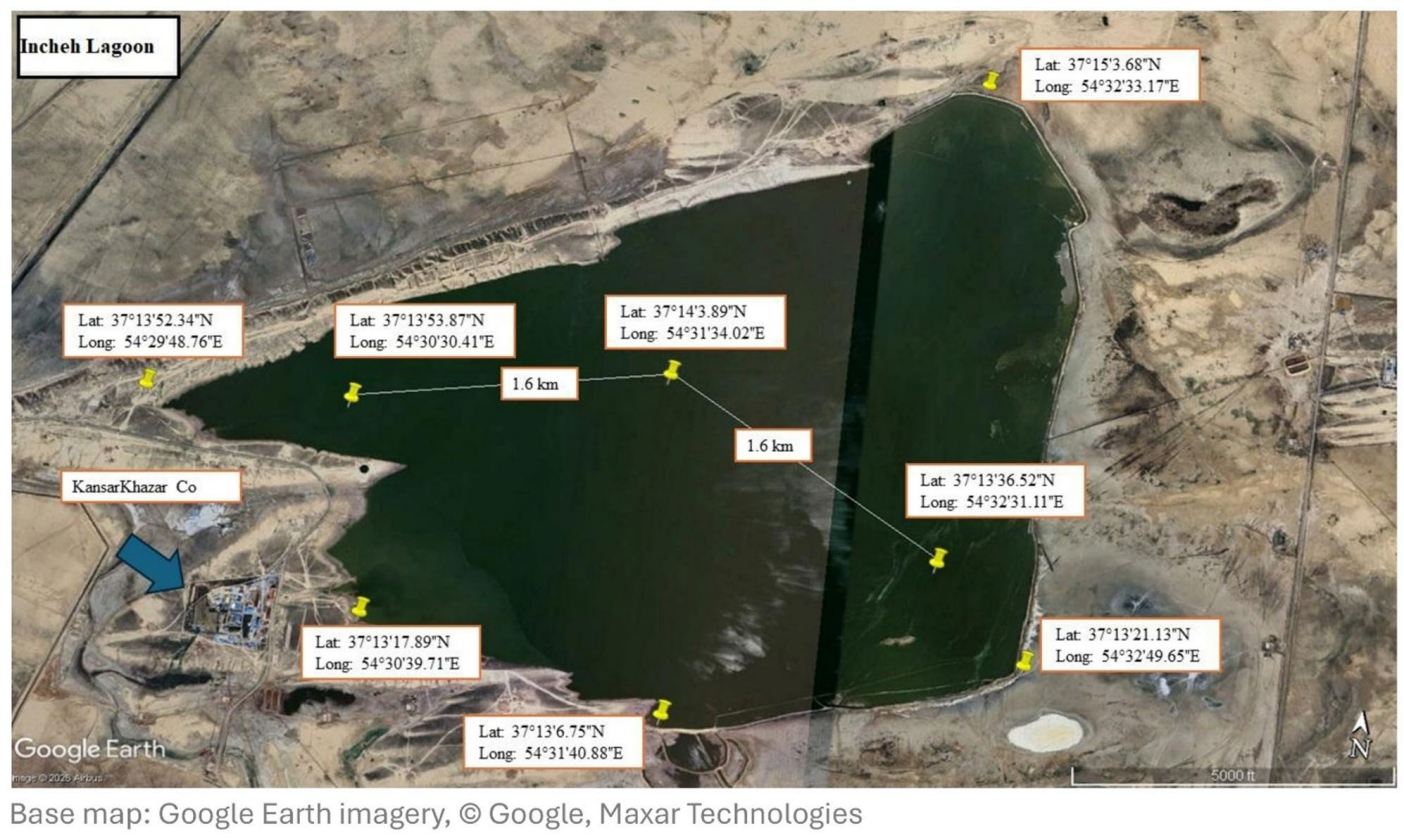
Study area map of the Incheh Lagoon, prepared using Google Earth imagery (Google, Maxar Technologies), 2024.

To delineate the basin and characterize its geomorphological features, topographic maps at a scale of 1:25,000 were employed. The map was prepared using Google Earth imagery (2024). According to hydrological observations, the lagoon’s water level exhibits significant seasonal fluctuations, ranging from + 3.5 m to −6.5 m relative to the reference datum (Fig. 1).

In-situ assessment of selected physicochemical parameters was conducted using portable multiparameter probes (WTW MultiLine Multi 3630 IDS, Weilheim, Germany). Water samples were collected in sterile polyethylene containers and preserved at 0 °C. Immediately after collection, water samples were placed in an icebox containing ice packs to maintain a temperature close to 0 °C during transport. This preservation method follows standard water-quality handling procedures recommended by APHA (2017), which advise cooling samples to ≤ 4 °C to prevent biological and chemical changes prior to laboratory analysis. All samples were delivered to the laboratory on the same day for physicochemical and metal analyses. Heavy metal concentrations were analyzed using Flame Atomic Absorption Spectrophotometry (FAAS; Shimadzu AA-7000, Kyoto, Japan), calibrated with certified standard solutions. The preserved samples were subsequently sent to certified analytical laboratories, including MABNA Laboratory in Tehran and the Environmental Research Laboratory at Gonbad University, for comprehensive evaluation. In total, 50 physicochemical parameters were analyzed across the collected water samples.

### Quality assurance and quality control (QA/QC)

Standard Quality Assurance and Quality Control (QA/QC) procedures were applied throughout sampling and laboratory analysis. Water samples were collected in pre-acid-washed polyethylene containers, transported at ≤ 4 °C, and accompanied by field blanks to monitor potential contamination. All reagents were of analytical grade, and glassware was acid-washed before use. Heavy metal measurements were performed using FAAS, and instrument calibration was carried out with certified multi-element standard solutions (Merck, Germany) covering the full analytical range of each metal. Analytical accuracy and precision were verified using reference materials and duplicate measurements. These procedures ensured the reliability and reproducibility of all reported metal concentrations.

The contamination factor (CF), which follows the classification framework proposed by Hakanson (1980), was used to quantify the extent of heavy metal contamination. This index facilitates the assessment of anthropogenic pollution in aquatic systems by comparing the observed concentrations with the established geochemical background values.

$$CF = \frac{{{C_{metal}}}}{{{C_{background}}}}$$

Where C_*metal*_ is the measured concentration of the metal in the sample, and C_*background*_ is the corresponding background or reference concentration. Hakanson’s classification^35^ scheme for CF is as follows:

1. Low contamination: CF < 1
2. Moderate contamination: 1 ≤ CF < 3
3. Considerable contamination: 3 ≤ CF < 6
4. Very high contamination: CF≥ 6

These classifications facilitate the assessment of the severity of heavy metal contamination in aquatic environments. At each sampling station, one liter of water was collected following the documentation of precise GPS coordinates. Phytoplankton identification and enumeration were conducted using polarized sunglasses to enhance visibility under field conditions. Samples were immediately fixed with 4% buffered formalin to preserve cellular integrity prior to laboratory analysis. In the laboratory, samples were homogenized and transferred into graduated cylinders for sedimentation over a 24-hour period, shielded from direct sunlight to prevent photodegradation. For samples exhibiting high turbidity or biomass concentration, a 1:10 dilution was performed using distilled water. The upper 500 mL was decanted, and the remaining volume was centrifuged at 3,000 rpm for 5 min. The supernatant was discarded, and the sediment was subjected to a second centrifugation cycle. Sediments from all tubes were pooled and resettled to a final volume of 5 mL. A 2 mL aliquot was analyzed using an inverted microscope at 40× magnification following a 6 h stabilization period. Taxonomic identification was performed using standard reference materials^36^^[,37^. For quantitative analysis, individual cells were counted in filamentous taxa, while colonial taxa were enumerated as discrete units. When cell density exceeded 3–4 cells per field at 20× or 40× magnification, a square ocular micrometer was used to standardize the counting area. A grid ruler was used to measure the side length of the micrometer and the diameter of the counting chamber, enabling accurate calculation of the observed area. Depending on cell density, 40 or 80 micrometer fields were counted to ensure a minimum of 100 cells per sample. For samples with low cell density or larger cell sizes, the entire chamber was analyzed. To minimize counting errors and enhance statistical reliability, the mean value of three independent chamber counts was calculated for each sampling station.

Zooplankton samples were collected using a 100 μm mesh plankton net with a 0.36 m mouth diameter, selected according to standard protocols for saline lagoon ecosystems to ensure efficient retention of *Artemia* and other dominant zooplankton taxa at three distinct sampling stations^38–40^.

The sampling area corresponds to the primary discharge zone of the iodine factory effluents, selected due to the highest likelihood of contamination and to accurately assess the environmental impact on the lagoon. A total of 400 L of water was filtered through the entire water column, divided into ten cycles of 40 L each. Samples were preserved in 4% buffered formalin and stored in 500 mL glass containers. In the laboratory, the total sample volume was recorded, homogenized thoroughly, and aliquots ranging from 100 to 350 mL were transferred into dish containers for microscopic examination. Under the microscope, fish eggs, larvae, and crab larvae were separated from the zooplankton samples. Zooplankton quantification was performed using mesh lamellae and randomly selected grid squares. The concentration of plankton per milliliter was calculated using the following formula:

$$\:NO./ML=\frac{C*1000{mm}^{3}}{A*D*F}$$

Where:

C: number of counted plankton; A: area of one grid square (mm²); D; depth of the field (mm); F = number of fields counted^41^.

Artemia samples were collected from the same saline lagoon stations using standardized plankton nets with a 100 μm mesh size, following internationally recognized protocols to ensure efficient retention of nauplii and adult individuals. Sampling was conducted across salinity gradients to capture population variability. Samples were preserved in 4% buffered formalin and processed under laboratory conditions similar to zooplankton, with aliquots examined microscopically for identification and quantification^42,43^.

Zooplankton density was calculated per unit volume based on the total volume of filtered water. Taxonomic classification was conducted to the family level using photographic records and standard identification guides. Family-level abundance was expressed in both liters and cubic meters, and seasonal variations across sampling stations were statistically analyzed. Aquatic macrophytes, recognized as key primary producers within the ecosystem, were sampled to evaluate their density using conventional field methods. At each station, an Ekman grab sampler was deployed to collect organic vegetation from the lagoon bed.

Macrobenthic invertebrates were collected using an Ekman grab sampler with a cross-sectional area of 400 cm². Samples were filtered through a 0.5 mm mesh sieve, preserved in 70% ethanol, and subsequently examined in the laboratory. Specimens were sorted and enumerated using multiple taxonomic identification guides, with species-level classification based on validated identification keys.

The primary objective of this study was to evaluate the current ecological status of the lagoon following 15 years of continuous industrial discharge. Anthropogenic activities have emerged as the dominant source of heavy metal contamination, particularly through industrial effluents, which significantly elevate metal concentrations in aquatic systems. The direct release of untreated wastewater from the iodine production facility into Incheh Lagoon presents a critical environmental concern. These findings are vital for assessing potential risks to aquatic biota and for informing the development of effective conservation and management strategies.

## Results

Measured concentrations of key heavy metals and physicochemical parameters demonstrated clear vertical and spatial variability within the lagoon. Manganese concentrations were 3.89 mg/L in deep water and 2.25 mg/L in surface water. Fluoride levels measured 4.75 mg/L in the industrial effluent, 4.6 mg/L in deep water, and 1.36 mg/L in surface water. Barium in the mining wastewater was 10.6 mg/L. Iron concentrations were 0.28 mg/L in surface water and 2.61 mg/L in deep water. Lithium levels were 0.42 mg/L in surface water and 0.6 mg/L in deep water. Bromine measured 0.47 mg/L in surface water. Nickel, zinc, and aluminum concentrations in surface water were 0.26 mg/L, 0.038 mg/L, and 0.26 mg/L, respectively.

The spatial variation in fluorine concentrations across different zones of the lagoon presents a noteworthy pattern. Elevated levels observed in the industrial effluent and deeper water layers suggest a potential accumulation over time, likely driven by continuous input and limited vertical mixing. In contrast, the reduced concentrations detected in surface waters may indicate dilution effects or the influence of alternative removal mechanisms such as volatilization, adsorption, or biological uptake. This distribution underscores the importance of assessing vertical profiles when evaluating water quality in stratified aquatic systems. Although iron and sulfide concentrations were found to be below regulatory thresholds, their role in the lagoon’s biogeochemical processes remains critical. These constituents can modulate the speciation, mobility, and toxicity of other trace metals through various chemical interactions. For example, iron is known to form stable complexes with several heavy metals, potentially reducing their bioavailability and mitigating their ecological impact on aquatic organisms. It is essential to acknowledge that even trace concentrations of certain metals can exert significant ecological effects, particularly in sensitive or enclosed aquatic environments.

The potential for bioaccumulation of heavy metals within the food web poses a significant ecological threat, particularly to higher trophic levels such as avian species that forage within the lagoon. The influx of industrial effluents into the Incheh Lagoon raises serious concerns regarding long-term impacts on water quality and overall ecosystem integrity. While the current assessment of metal concentrations in the water column provides valuable baseline data, it underscores the necessity of establishing long-term monitoring programs to track temporal and seasonal variations. Such initiatives are essential for detecting emerging trends and understanding the cumulative effects of industrial discharges. The unique physicochemical characteristics of the Incheh Lagoon, namely its elevated salinity and the presence of halotolerant organisms such as *Artemia*, further complicate the interpretation of these findings. This observation aligns with established literature indicating that salinity levels above 90 ppt can impair reproductive performance in *Artemia*^43^^[,44^. High salinity levels can alter the speciation and bioavailability of metals, potentially modulating their toxicity depending on the specific metal and prevailing environmental conditions. Continued investigation into the biogeochemical behavior of heavy metals within this ecosystem is imperative. A sustained research effort will enhance our understanding of the lagoon’s ecological dynamics and support the development of targeted conservation and management strategies. Ultimately, this study contributes to the broader field of aquatic ecotoxicology by offering insights applicable to similarly impacted ecosystems subjected to industrial stressors (Table 1).

**Table 1:** Standard values are based on the effluent discharge standards of the Iran Department of Environment (DOE).

Parames	Standard^**^	Influent water to factory	Efluent water to Incheh	Surface water to Incheh	Pollution Class	Deep water to Incheh	Pollution Class	Surface water adjucen lagoon
As (mg/L)	0.1	> 0.005	-	> 0.02	low	-	-	> 0.1
Hg (mg/L)	rare	> 0.001	> 0.002	0.011	low	> 0.002	low	> 0.05
Pb (mg/L)	1	> 0.005	> 0.1	0.055	low	> 0.1	low	> 0.05
Cd (mg/L)	0.1	0.13	0.007	0.00385	low	> 0.007	low	> 0.003
Cu (mg/L)	1	0.19	> 0.03	0.0165	low	> 0.03	low	> 0.015
Fe (mg/L)	3	4.2	1.3	1.745	low	2.61	low	0.28
V (mg/L)	0.1	0.1	> 0.02	0.011	low	> 0.02	low	> 0.01
Cr (mg/L)	2	0.21	> 0.01	0.007	low	> 0.01	low	0.03
Mn (mg/L)	1	0.63	3.2	2.25	moderate	3.89	considerable	> 0.46
Li (mg/L)	2.5	0.2	0.58	0.42	low	0.6	low	0.26
Co (mg/L)	1	1.07	> 0.01	0.0055	low	> 0.01	low	> 0.025
Ag (mg/L)	1	0.31	0.04	> 0.024	low	0.04	low	> 0.02
Ni (mg/L)	2	1.1	> 0.03	> 0.0165	low	> 0.03	low	> 0.015
Al (mg/L)	5		> 0.1	0.26	low	> 0.1	low	> 0.07
Ba (mg/L)	5	100	10.6	3.54	low	4.89	low	0.15
Zn (mg/L)	2	0.21	> 0.07	0.038	low	> 0.07	low	> 0.06
Mo (mg/L)	0.01	> 0.05		> 0.002	low			> 0.01
Se (mg/L)	-	> 0.005	> 0.1	0.063		> 0.1		> 0.14
B (mg/L)	-	4.2		7.06				20.95
Be (mg/L)	0.1	> 0.1	> 0.004	> 0.002	low	> 0.004	low	> 0.002
Cyanide (mg/L)	0.5		> 0.04	> 0.02	low	> 0.029	low	> 0.03
Phenol (mg/L)	1		> 0.05	> 0.05	low	> 0.05	low	> 0.05
Sulfide (mg/L)	3	> 0.05	0.16	> 0.15	low	> 0.2	low	> 0.03
Sulfites (mg/L)	1		> 0.1	> 0.1	low	> 0.1	low	> 0.1
Br (mg/L)	2	4.2						0.47
F (mg/L)	2.5	> 1	4.75	1.36	low	4.6	moderate	2.55
G&O	10	> 1		> 10	low	> 10	low	> 10
Color	75	2		19.39	low	19.39	low	
Detegent	1.5			1.37	low	1.56	moderate	

In comparing the physicochemical characteristics of the Incheh Lagoon with established standards for surface water and reference values from Lake Urmia, where *Artemia* serves as a bioindicator species, our observations revealed that pH and sulfate (SO₄²⁻) levels in the lagoon remained within the acceptable range for the survival and growth of *Artemia* and algal communities. Most other parameters also fell within biologically tolerable limits, with the exception of calcium and magnesium, which exhibited elevated concentrations. Relative to the adjacent lagoon, the Incheh Lagoon demonstrated increased levels of total dissolved solids (TDS), total suspended solids (TSS), calcium, and magnesium, while bicarbonate concentrations showed a noticeable decline. Notably, TSS concentrations in both surface and deep water samples were approximately three times higher than the standard threshold, indicating substantial particulate loading. Despite these elevated TSS levels, algal growth was not entirely inhibited. However, a marked reduction in the presence of blue-green algae was observed, suggesting that specific physicochemical conditions such as increased turbidity or ionic composition may selectively influence algal community structure and species dominance.

**Fig. 2:**
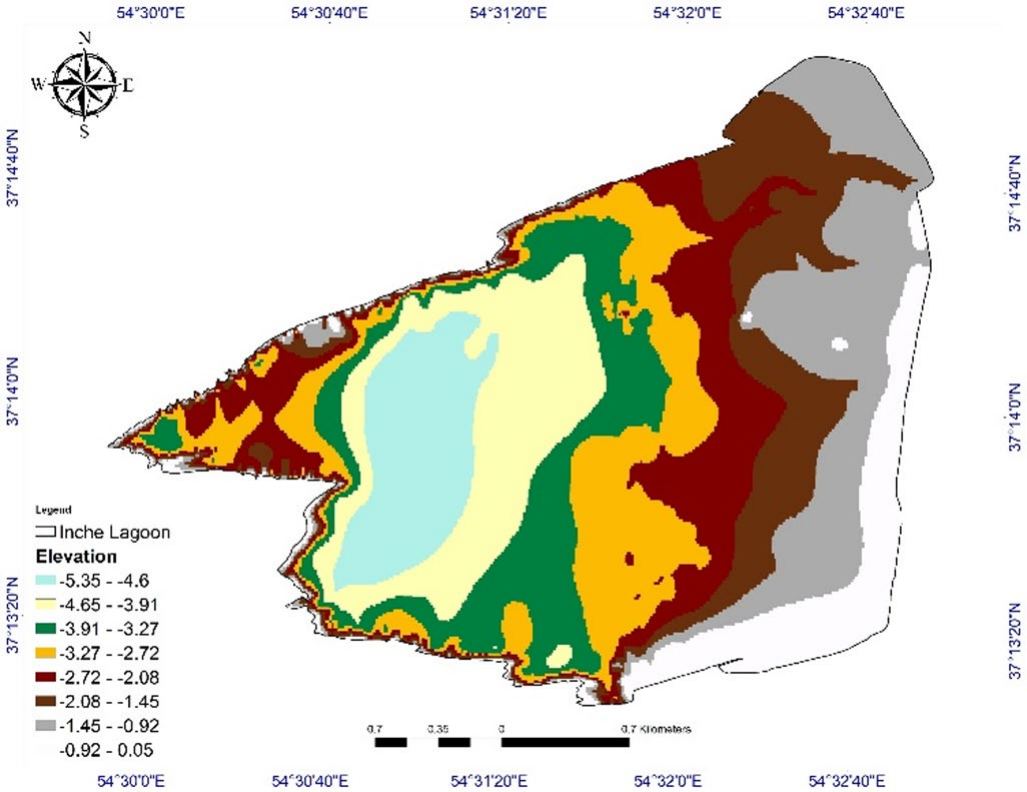
Physiographic condition of Incheh Lagoon - Golestan Province. The map was generated by the authors using QGIS version 3.28 (https://qgis.org) based on surveyed elevation points.

Figure 2 illustrates the physiographic condition of Incheh Lagoon in Golestan Province, which was generated using QGIS version 3.28 (https://qgis.org). The process began by importing surveyed elevation points into QGIS as a vector layer, incorporating their spatial coordinates and height data. These points served as control points for the subsequent interpolation process. Using the interpolation tools available within QGIS, specifically the Kriging or IDW (Inverse Distance Weighting) method, a digital elevation model (DEM) of the region was created, providing a continuous surface that reflects the area’s topography. Based on this DEM, contour lines were then delineated at specified elevation intervals to accurately represent the physiographic features of the lagoon area. Additional spatial analyses such as boundary delineation and feature mapping were performed to enhance the map’s clarity and detail. The final map was styled with appropriate color schemes, labels, and legends, and exported as a high-quality image for presentation purposes. This methodology ensured a precise and detailed representation of the physiographic conditions of Incheh Lagoon, facilitating further spatial and environmental analysis.

The pH level of the lagoon was slightly below the minimum environmental standard, rendering the water mildly acidic. Nevertheless, both algal communities and *Artemia* populations continue to thrive within this pH range, indicating a degree of tolerance to suboptimal conditions. As a hypersaline wetland, the salinity of Incheh Lagoon fluctuated between 95 and 200 g per liter during the autumn and winter seasons. Historical data prior to the onset of industrial activity indicated a broader salinity range, spanning from 95.3 to 295 ppt. Occasionally, salinity levels dropped below 40 g per liter, creating favorable conditions for the hatching of *Artemia* cysts. However, the continuous discharge of industrial wastewater currently sustains salinity levels above 90 g per liter (even during wetter seasons) thereby impeding the emergence of *Artemia* from dormant cysts and potentially disrupting their reproductive cycles.

Biochemical Oxygen Demand (BOD) levels in the lagoon’s water column were markedly below environmental standards, indicating low levels of biodegradable organic matter. In contrast, nitrate (NO₃⁻) concentrations were significantly elevated (approximately 13 times higher than the permissible limit), suggesting potential nutrient enrichment likely linked to anthropogenic inputs.

Water hardness exhibited extreme elevation, measuring nearly twice that of local surface water and 1.5 times greater than pre-industrial reference values. Calcium concentrations were notably high, reaching approximately 3.6 times the levels recorded in the surface water of the adjacent lagoon. Similarly, magnesium levels exceeded standard thresholds by approximately 1.7 times compared to nearby surface waters and were 13.5 times higher than pre-industrial benchmarks. Potassium concentrations were elevated but remained lower than those observed in the surface water of the neighboring lagoon. Sulfate (SO₄²⁻) levels surpassed permissible limits, yet were comparable to those found in adjacent surface waters, indicating a regional pattern of elevated sulfate concentrations. Sodium and chloride levels were also elevated, measuring approximately 1.5 and 2 times higher, respectively, than those in the nearby lagoon’s surface water. Despite these ionic imbalances, the abundance of *Escherichia coli* remained significantly below standard values (Table 2).

**Table 2:** Physicochemical characteristics of water samples from the Incheh Lagoon and adjacent areas.

Parameters	Influent water to factory	Surface water (Incheh)	Deep water (Incheh)	Surface water adjacent (lagoon)	Influent water to factory	Incheh Hami Tabari^34^,	Urmia Lake
pH	6.5–8.5	6.16	6.32	7.73	6.85	7.5–8.6	-
EC (mS/Cm)	-	127.2	130.2	178.4	140	155	-
DO (mg/l)	-	8.22	7.97	-	0.1	7.49	-
DO%	-	96.8	93.6	-	-	-	-
Salinity (ppt)	-	164.3	166.1	116.4	65	138.3 95.3–295 (40)	-
TDS (g/l)	-	152/5	160/3	112/4	91	-	-
TSS (g/l)	60	188.3	183.7	41.92	25	-	-
TS (g/l)	-	340.8	344	154.32	-	-	-
BOD (mg/l)	50	3.79	3.75	-	19	5.1	-
NO_3_ (µg/l)	50,000	400	635	600	51	-	-
NO_2_ (µg/l)	10,000	10.35	8.28	19	0.05	-	-
Hardness (g/l)	-	76.3	75.6	36	40	48.1	-
Ca (g/l)	0.075	27.08	27	7.5	10	0.55	2
Mg (g/l)	0.1	49.2	48.58	28.5	3.6	10.5	25
K (mg/l)		86	76.8	106	131	-	6
SO_4_^2−^ (mg/l)	400	486.5	428.8	485	5	-	45,000
HCO_3_^−^ (mg/l)		35	33.3	125		139.5	-
Na^+^ (g/l)	1	96.9	99.6	70		-	-
Cl^−^ (g/l)	1	122.6	155.9	71		-	-
T.*EcoliMPN*/100 ml	1000	119.2	92.2	74		-	-

^**^Standard of wetlands (approved by the Iranian Board of Ministers, 2021).

The elevated concentrations of nitrate (NO₃⁻), calcium, and magnesium indicate significant disruptions in the nutrient and mineral balance of the Incheh Lagoon. These shifts may exert wide-ranging ecological effects, potentially favoring certain species while disadvantaging others, thereby altering community composition and trophic interactions. Such findings underscore the complex interplay between natural processes and anthropogenic influences in shaping the lagoon’s water chemistry. The presence of industrial effluents raises concerns about long-term degradation of water quality and the resilience of aquatic ecosystems. Moreover, the persistently high salinity levels (exacerbated by continuous sewage discharge), complicate the geochemical behavior of metals. Elevated salinity can affect metal speciation and bioavailability, potentially amplifying or mitigating toxicity depending on the specific element and prevailing environmental conditions.

This sustained salinity presents a critical challenge to *Artemia* populations, a keystone component of the lagoon’s food web. Impaired hatching and reproductive success due to unfavorable salinity conditions may disrupt ecological stability and reduce the lagoon’s biological productivity.

During field investigations, remnants of partially decomposed pasture vegetation were identified at the bottom of the wetland, specifically at Station 2 located in its central region. This observation contributes to a deeper understanding of the ecological processes within the Incheh Lagoon and offers valuable insights for the management of similar aquatic systems subjected to industrial pressures. Phytoplankton analysis revealed low species richness and density, with only nine genera identified: *Chroococcus*, *Oscillatoria*, *Ankistrudesmus*, *Euglena*, *Prorocentrum*, *Gymnodinium*, *Amphiprora*, *Nitzschia*, and *Synechococcus*. Among these, only *Synechococcus* a genus of cyanobacteria exhibited low to moderate abundance, while the remaining genera appeared sporadically and in minimal quantities. Comparative analysis with previous studies highlights temporal consistency in phytoplankton composition. In 2018, six genera from four distinct phyla were reported, whereas a 2021 study identified nine genera spanning five phyla. It is important to note that the apparent increase in taxonomic representation does not necessarily indicate enhanced biodiversity; rather, it likely reflects improvements in sampling techniques and analytical resolution. Despite these methodological differences, the Incheh Lagoon consistently demonstrates low phytoplankton diversity and biomass, suggesting that environmental constraints such as elevated salinity, nutrient imbalances, and industrial influence may be limiting primary productivity and shaping community structure (Table 3).

**Table 3:** Abundance of identified phytoplankton (per liter) in Incheh lagoon.

Phylum	Genus	2018	2023
Cyanobacteriota	*Synechococcus*		10,444,791
	*Chroococcus*		25,883
	*Oscillatoria*		50,625
	*Anabaena*	900	
Chlorophyta	*Ankistrudesmus*		667,500
	*Clamydomonas ovalis*	1200	
Euglenophyta	*Euglena*	600	1458
Dinoflagellata	*Prorocentrum*		3125
	*Gymnodinium*		7708
	*Amphiprora*	1500	417
	*Nitzschia*		17,083
Bacillariophyta	*Gyrosigma*	7000	
	*Navicula*	4500	
	*Synedra*	600	

The zooplankton assessment revealed a limited faunal diversity, with only five genera identified throughout the sampling process. Quantitative analysis conducted on 400 L of filtered water indicated extremely low zooplankton abundance, as detailed in Table 4. This scarcity suggests a potentially stressed or nutrient-limited environment, possibly influenced by elevated salinity, industrial discharge, or other physicochemical constraints.

**Table 4:** Average relative abundance of detected zooplankton (number per liter) of Incheh lagoon.

Phylum	Genus	2018	2023
Ciliophora	*Tintinnopsis*	-	0.025
	*Keratella*	2.5	-
Amoebozoa	*Difflugia*	-	0.022
	*Arcella*	0.75	0.003
Arthropoda	*Ostracod*	-	0.002
Sarcomastigophora	*Foraminifera*	-	0.003

A particularly noteworthy finding of this study is the complete absence of *Artemia* species within the Incheh Lagoon. This result contrasts with earlier research by Hami Tabari et al. (2006), which documented the presence of *Artemia* from December through April. Subsequent investigations in 2012 and 2017 similarly failed to detect *Artemia* at any sampling stations, suggesting a long-term decline or disappearance of this keystone species.

These observations point to substantial alterations in the lagoon’s planktonic community structure over time. The consistently low diversity and abundance of both phytoplankton and zooplankton reflect a stressed and potentially degraded ecosystem. The disappearance of *Artemia*, a species integral to the lagoon’s trophic dynamics, raises serious ecological concerns. Benthic assessments further support this trend, revealing minimal faunal diversity. Only two molluscan groups snails and gastropods were identified, indicating limited benthic resilience and reduced habitat complexity.

The observed ecological shifts may be indicative of changing environmental conditions, potentially linked to industrial activities and modifications in water chemistry previously discussed. To elucidate the underlying causes of these community transformations, future research should prioritize identifying the key factors constraining plankton diversity and abundance. Implementing long-term monitoring programs that integrate planktonic community assessments with water quality measurements will be essential. Such efforts could yield critical insights into the lagoon’s ecological trajectory and support the development of informed conservation and management strategies.

## Discussion

The recent ecological changes in the Incheh Lagoon appear to be primarily due to anthropogenic stressors, particularly industrial discharge and hydrological inputs changes that have significantly impacted water chemistry and disrupted plankton dynamics. Long- term studies in Lavaca Bay and other semi-enclosed coastal systems have shown that industrial effluents can lead to persistent shifts in plankton community structure, often favouring stress-resistant taxa and reducing overall biodiversity. In addition, increased salinity and nutrient variability, the hallmarks of hypersaline environments, have been shown to affect plankton productivity and alter elemental stoichiometry, particularly under conditions of nutrient limitation and osmotic stress^45^.

Given the pivotal role of plankton as basal components of aquatic food webs and sensitive indicators of ecosystem integrity, elucidating the mechanisms underlying biodiversity loss is imperative. Recent findings suggest that salinity-driven changes in zooplankton assemblages can diminish grazing pressure on phytoplankton, thereby exacerbating eutrophication and destabilizing trophic interactions^46^. To address these challenges, future research should adopt integrative frameworks that combine high-resolution water quality assessments with long-term plankton monitoring. Such approaches have proven effective in other lagoon systems, where continuous plankton recorder data revealed significant trends linked to climatic and anthropogenic pressures^47^. Implementing adaptive conservation strategies such as regulating industrial discharge thresholds, restoring hydrological connectivity, and enhancing biomonitoring protocols, will be essential for safeguarding the lagoon’s ecological resilience in the face of accelerating environmental change^48^.

The ecological trajectory of Incheh Lagoon has undergone significant transformations over the past decades. Prior to the onset of industrial activities and the discharge of wastewater, the lagoon exhibited seasonal variability and was not a permanent water body. Periods of low precipitation combined with elevated evaporation rates often resulted in complete desiccation during summer months. Historically, inflow from the Voshamgir Dam enriched the lagoon with nutrients and moderated salinity levels. Episodic rainfall events in spring and summer temporarily reduced salinity to approximately 30 ppt, facilitating the hatching of *Artemia* cysts. However, these favorable conditions were short-lived and insufficient to sustain robust *Artemia* populations. The subsequent introduction of hypersaline effluents (exceeding 150 ppt) disrupted cyst viability and led to the local extinction of *Artemia*. Nevertheless, strategic regulation of effluent salinity could potentially restore *Artemia* viability, given their tolerance threshold below 150 ppt.

A comparative assessment of Incheh Lagoon’s water quality against environmental standards reveals a highly stressed aquatic ecosystem. The primary limiting factors for biodiversity include persistently elevated salinity levels (> 150 ppt), low pH values approaching the biological tolerance threshold (~ 6), and increased concentrations of manganese (Mn), calcium (Ca), and magnesium (Mg). These conditions impair physiological functions in aquatic organisms and compromise ecosystem resilience. To restore ecological integrity, a comprehensive strategy integrating hydrological and hydrobiological rehabilitation is essential. Hydrological restoration should focus on increasing freshwater inflow from regional sources, including surplus discharge from the Voshmir Dam and agricultural runoff, potentially contributing between 32 and 93 L per second. This influx could help dilute salinity and rebalance ionic composition. Manganese, while essential in trace amounts, poses significant ecological risks when present in excess. Elevated Mn concentrations can induce oxidative stress in aquatic plants, disrupt nutrient uptake, and impair photosynthetic efficiency^49^. Recent studies have demonstrated that Mn-rich ores can enhance the sorption and retention of both organic and inorganic compounds through organo- mineral- microbe interactions, particularly in sediment and wetland systems^50^. Moreover, manganese oxides have shown high efficacy in constructed wetlands, improving pollutant removal and supporting microbial processes critical to water purification^51,52^. In addition, bioaccumulation of Mn in aquatic vegetation has been linked to potential toxicity and disruption of trophic dynamics in wetland ecosystems^53^.

The primary sources of manganese (Mn) in wetland environments include natural geological deposits, industrial heavy metal discharges, and deliberate additions for remediation purposes. The redox cycling between Mn(II) and Mn(IV) plays a pivotal role in enhancing pollutant removal in constructed wetlands, particularly through microbial-mediated oxidation and reduction processes. Mn-based substrates have demonstrated high efficiency in reducing ammonia-nitrogen (NH₄⁺-N), organic contaminants, and heavy metals such as cadmium (Cd) and lead (Pb), offering viable solutions for complex wastewater treatment challenges^54,55^. To comprehensively evaluate system performance, both internal factors (microbial community composition, plant species, pH levels) and external drivers (seasonal temperature fluctuations, rainfall variability, UV radiation) must be considered. Among these, microbial activity, pollutant load, and temperature have emerged as key determinants of wetland functionality^17^. When applying Mn-enriched matrices in constructed wetlands, it is essential to account for the tolerance thresholds of associated plant species. Experimental findings from Mn-polluted wetland trials revealed low survival rates for *Hydrocotyle vulgaris*, and growth inhibition in *Arundo donax* and *Acorus calamus*. In contrast, species such as *Thalia dealbata*, *Boehmeria nivea*, *Canna indica*, *Phragmites australis*, *Typha orientalis*, *Nerium oleander*, *Pontederia cordata*, *Scirpus validus*, and *Iris germanica* exhibited robust growth and adaptability. These resilient taxa have been successfully implemented in leachate collection and treatment systems for Mn-contaminated soils, as demonstrated in the Xiangtan Manganese Mine restoration project^3^.

It is noteworthy that although iron underwent oxidation in the wetland, there was no significant decrease in pH. The hydrolysis of iron generates hydrogen ions, which may cause a reduction in pH. Nonetheless, it is clear that the environment possesses a buffering capacity linked to the carbonate chemistry of the water, which stops this pH decrease. Recent studies confirm that the CO₂ - carbonate system plays a crucial role in maintaining pH stability in aquatic environments, especially during redox reactions involving metals such as iron^56^. Iron can be effectively removed from mine water by implementing passive treatment systems like oxidation ponds and wetlands. The oxidation of iron seems to be the key removal mechanism in the initial stages of the system. Nonetheless, when iron levels drop to a certain point, biological removal processes might gain increased significance. Microbial iron oxidizers and wetland vegetation have been shown to enhance iron attenuation under low concentration conditions^57^.

In addition, research is required to assess the comparative significance of various biota in the elimination of iron in wetlands. Manganese can also be eliminated through the use of wetlands. It is evident that iron needs to be eliminated before the removal of manganese can occur, as manganese oxidation is slower and often biologically mediated. Recent findings indicate that manganese removal efficiency is influenced by factors such as water temperature, iron particle concentration, and microbial activity^58,59^. Furthermore thorough observation and analysis of the significance of both biotic and abiotic removal processes that take place over time and space in wetlands will enable engineering and management choices to enhance and guarantee the ongoing efficiency of removal systems. Constructed wetlands, when properly designed with attention to hydrology, substrate composition, and vegetation, can harness both microbial and physicochemical mechanisms to sustain long-term metal removal^60^.

Regrettably, for many communities worldwide, avoiding fluoride-contaminated water is not a viable option due to limited access to alternative sources. To address this challenge, a range of in-situ and ex-situ remediation techniques have been developed to reduce fluoride concentrations in groundwater to acceptable levels. These include adsorption, membrane filtration, ion exchange, electrocoagulation, and emerging bioadsorption methods using agricultural waste-derived materials^61^. Managing fluoride contamination and mitigating fluorosis remain critical concerns for drinking water safety. Priority should be placed on the use of alternative water supplies or blended sources, particularly in regions with endemic fluoride exposure. In parallel, emphasis must be placed on the development of robust, scalable technologies and integrated treatment systems that are not only efficient and cost-effective but also adaptable to local socio-economic and environmental conditions^62^.

Moreover, ongoing research into defluoridation agents and novel materials such as modified biochar, nanocomposites, and hybrid adsorbents continues to advance fluoride remediation strategies^61,63^. These innovations aim to improve selectivity, regeneration potential, and environmental compatibility, offering scalable soloutions for contaminated water systems. In summary, fluoride contamination in groundwater represents a complex challenge with profound implications for both human health and ecological stability. Recent studies have highlighted its dual origin of fluoride contamination: natural geogenic sources such as fluorite-bearing lithologies and anthropogenic activities including industrial effluents, fertilizer runoff, and coal combustion byproducts^64^. The comprehensive analyses of fluoride origins confirm that both natural geological processes and human activities play a role in the presence of fluoride in groundwater systems (Roshni and Harikumar, 2021).

## Conclusion and recommendations

Human activities in and around these sensitive environments are increasingly threatening lagoon ecosystems. In the case of Incheh Lagoon, contamination by heavy metals particularly manganese and fluorine originates from anthropogenic sources such as wastewater discharge from an iodine production facility. These findings underscore the complex interplay of factors influencing the lagoon’s ecological dynamics. Moving forward, a comprehensive management strategy must account for these multifaceted stressors. Recommended actions include:

1. Salinity Regulation: Implement measures to reduce and regulate effluent salinity to levels that support aquatic life.
2. Water quality improvement: Tackling additional water quality concerns, particularly acidity and elevated mineral concentrations.
3. Freshwater augmentation: Explore methods to increase freshwater inflow, therby reducing salinity and improving overall water quality.
4. Habitat restoration: Rehabilitate suitable habitats for key species such as *Artemia* and various plankton communities.
5. Continuous monitoring: Establish a long-term monitoring program to track changes in water quality, plankton populations, and benthic organisms over time.

## Supplementary Information

Below is the link to the electronic supplementary material.

Supplementary Material 1

## Data Availability

The datasets generated and/or analyzed during the current study are not publicly available; however, they may be obtained from the corresponding author upon reasonable request.
