# Supplementary material for: Impacts of iodine factory effluents on the water quality and ecosystem health of Incheh Lagoon in Iran
Source: Sci Rep. 2026 May 16;16:22223. doi: 10.1038/s41598-026-52359-0 (PMC13369748; doi:10.1038/s41598-026-52359-0)
Supplement: Supplementary file 1 — Supplementary Material 1 [file 41598_2026_52359_MOESM1_ESM.pptx]

## Slide 1
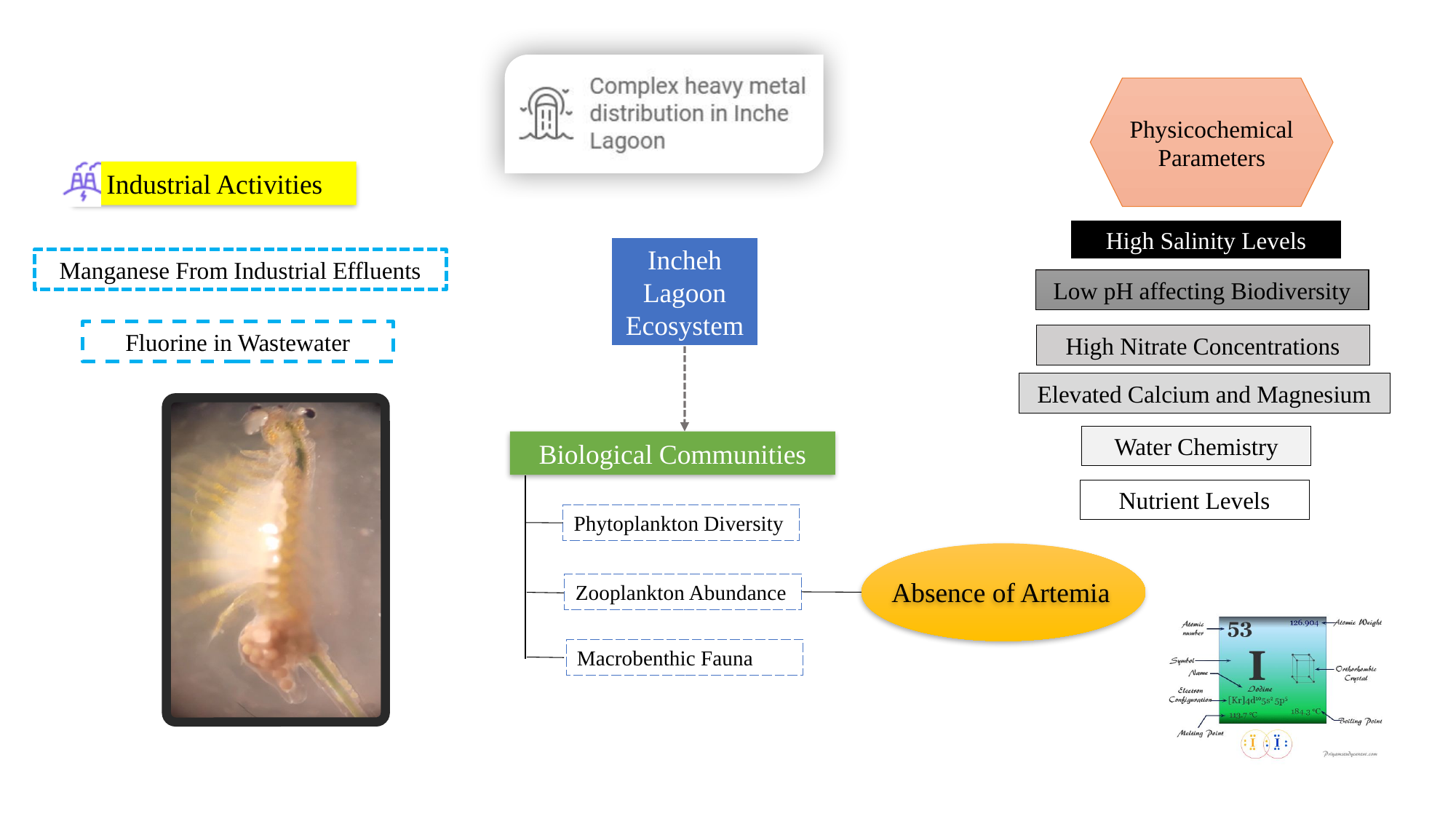

Physicochemical Parameters
Industrial Activities
High Salinity Levels
Incheh Lagoon Ecosystem
Manganese From Industrial Effluents
Low pH affecting Biodiversity
Fluorine in Wastewater
High Nitrate Concentrations
Elevated Calcium and Magnesium
Water Chemistry
Biological Communities
Nutrient Levels
Phytoplankton Diversity
Absence of Artemia
Zooplankton Abundance
Macrobenthic Fauna
